# Characteristics of Morphology in Older Adult Patients with Obstructive Sleep Apnea: A Retrospective Cross-Sectional Study

**DOI:** 10.3390/healthcare13172190

**Published:** 2025-09-02

**Authors:** Liqin Wang, Keishi Wada, Kentaro Okuno, Akio Himejima, Ayako Masago, Kazuya Takahashi

**Affiliations:** 1 Department of Geriatric Dentistry, Osaka Dental University, 8-1, Kuzuhahanazonocho, Hirakata 573-1121, Japan; wang-l@cc.osaka-dent.ac.jp (L.W.); wada-k@cc.osaka-dent.ac.jp (K.W.); masago-a@cc.osaka-dent.ac.jp (A.M.); kazuya-t@cc.osaka-dent.ac.jp (K.T.); 2 Center for Dental Sleep Medicine, Osaka Dental University Hospital, 1-5-17, Otemae, Chuo-ku, Osaka 540-0008, Japan; 3 Department of Geriatric Dentistry, Osaka Dental University Hospital, 1-5-17, Otemae, Chuo-ku, Osaka 540-0008, Japan; 4 First Department of Oral and Maxillofacial Surgery, Osaka Dental University, 1-5-17 Ohtemae, Chuo-ku, Osaka 540-0008, Japan; himejima@cc.osaka-dent.ac.jp

**Keywords:** obstructive sleep apnea, geriatric, upper airway

## Abstract

**Objective:** The prevalence and severity of obstructive sleep apnea (OSA) increase with age, suggesting that age-related factors are etiological factors for OSA in older adults. In addition to anatomic contractions of the upper respiratory tract, such as those caused by obesity and retrognathia, sleep is impaired in older OSA patients due to aging. Although aging has long been associated with structural changes in the upper airway potential, specific age-related anatomical differences in patients with OSA are not established. This study aimed to examine age-related morphological differences in OSA patients, particularly in older adults. **Methods:** This study was designed as a retrospective cross-sectional study conducted at the Center for Dental Sleep Medicine, Osaka Dental University, between May 2017 and September 2022. From an initial cohort of 1032 patients, 183 male participants were included after applying strict inclusion and exclusion criteria. Patients were classified into two age groups: middle age (40–60 years) and older age (≥65 years). Polysomnographic parameters; body mass index (BMI); airway space (AS) obtained from cephalometric radiographs; length of the soft palate (PNS-P); SNB angle, as an indicator of mandibular position; and the position of the hyoid bone (MP-H) were compared between the groups. Statistical analysis included Levene’s test for homogeneity of variances, independent sample *t*-tests for group comparisons, and multiple regression analyses to identify independent predictors of AHI. This study was conducted with the approval of the Ethics Committee of Osaka Dental University (No. 111047). **Results:** Older patients showed significantly lower REM sleep percentage (13.5 ± 1.31% vs. 16.4 ± 0.59%, *p* = 0.047), significantly lower BMI (23.6 ± 0.45 kg/m^2^ vs. 24.6 ± 0.29 kg/m^2^, *p* = 0.049), and significantly larger AS (15.8 ± 0.52 mm vs. 12.0 ± 0.27 mm, *p* = 0.000) compared to middle-aged patients. Furthermore, in the middle-aged group, BMI (β = 0.40, 95% CI: 1.46 to 3.41, *p* < 0.001), SNB (β = −0.18, 95% CI: −1.75 to −0.09, *p* = 0.030), and MP-H (β = 0.19, 95% CI: 0.10 to 1.01, *p* = 0.018) were significant independent predictors of AHI. In the older group, no parameters were significant predictors of AHI. **Conclusions:** We found that older adult patients had a larger airway diameter and lower REM sleep percentage and BMI than middle-aged patients. Furthermore, regarding factors associated with AHI, which is an indicator of sleep apnea severity, in the middle-aged group, anatomical factors such as BMI, SNA, and MPH contributed significantly, but in the older adult group, anatomical factors were not relevant. The results suggested that anatomical factors alone may not fully explain the pathogenesis of OSA in older patients, highlighting the need for further studies focusing on other age-related factors.

## 1. Introduction

Obstructive sleep apnea (OSA) is one of the more common forms of sleep-disordered breathing. OSA is characterized by recurrent episodes of upper airway obstruction during sleep resulting in intermittent hypoxia and sleep fragmentation. OSA is characterized by recurrent episodes of upper airway obstruction during sleep. Sleep fragmentation and intermittent hypoxia result in sympathetic activation, systemic inflammation, and oxidative stress, which, all together, significantly increase cardiometabolic risk [1,2]. The strongest risk factors for OSA are obesity and a high BMI index, but other factors include male gender and age over 50 years. With its prevalence increasing with age, OSA has become a major health concern in older adults [3,4,5].

In middle-aged populations, OSA is mainly associated with morphological factors such as obesity, retrognathia, and micrognathia [6,7,8,9]. These factors contribute to narrowing and obstructing the upper airway during sleep. Furthermore, recent studies have reported that non-anatomical factors, such as ineffective pharyngeal dilator muscle function during sleep, a low threshold for arousal to airway narrowing during sleep, and unstable control of breathing (high loop gain), also contribute to the development of OSA [10]. Although these morphological factors are recognized to play a role in OSA in middle-aged patients, their specific role in older adults remains to be elucidated [11,12]. A recent study [13,14,15] reported that the age-related increase in OSA prevalence and severity cannot be fully explained by morphological factors, despite these being common predisposing factors in middle-aged populations. The increased prevalence and severity of OSA with age indicate that factors beyond anatomical changes play a role in its development in older adults. The pathogenesis of OSA in older patients is more likely to be complex.

The purpose of this study was to investigate the morphological characteristics of OSA in elderly patients and to compare anatomical factors contributing to apnea severity with those in middle-aged patients.

## 2. Materials and Methods

### 2.1. Study Design and Setting

This study was designed as a retrospective cross-sectional study conducted at the Center for Dental Sleep Medicine, Osaka Dental University, between May 2017 and September 2022. The study design and reporting followed the STROBE guidelines.

### 2.2. Subjects

**Figure 1:**
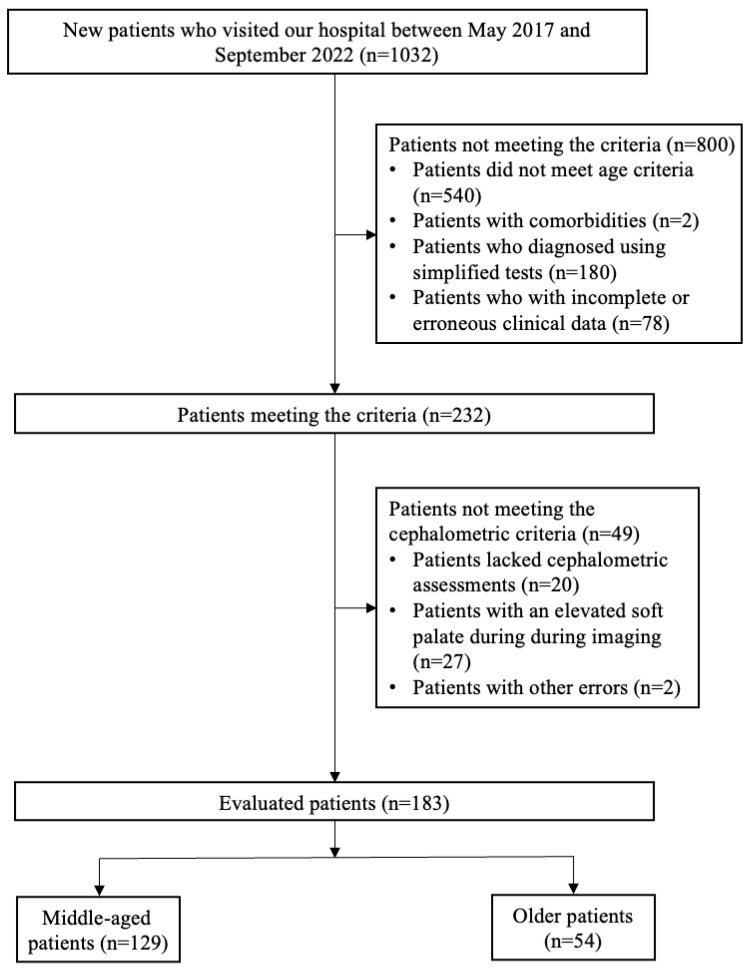
Flow diagram of the patient screening process. Patients (n = 183) were classified into three groups based on the severity of OSA, as measured by the age: middle age (40–60 years), older age (over 65 years).

Between May 2017 and September 2022, a comprehensive retrospective cohort study was conducted involving 1032 patients at the Center for Dental Sleep Medicine of our hospital. The inclusion criteria were (1) males only; (2) middle age (40 ≤ age < 60) or older (age ≥ 65); (3) apnea–hypopnea index (AHI) ≥ 5 determined through polysomnography; and (4) undergoing cephalometric assessment. Although conducting separate analyses by sex would have been preferable, the small number of eligible female patients made this approach unfeasible. Bixler et al. [16] reported that menopause is a significant risk factor for OSA in females, with hormone replacement therapy potentially mitigating this risk. Males were selected because of a lack of female patients and the pathophysiology of OSA differs between the sexes. Exclusion criteria included patients who did not meet the age criteria, specifically if they were younger than 40 years or between 60 and 64 years. To minimize transitional overlap, participants aged 60–64, which may overlap with the characteristics of both middle age and older age people, were excluded. And patients with comorbidities that could significantly affect sleep quality and airway structure (e.g., tumors in the maxillofacial region, severe heart disease, cerebrovascular disease, or neuromuscular disease) and those diagnosed using an out-of-center sleep test rather than full polysomnography. Data-cleaning procedures were applied to remove patients with incomplete or erroneous clinical data, including missing BMI or AHI values. Following the application of exclusion criteria, 800 patients who did not meet these criteria were removed from the cohort, leaving 232 patients for further evaluation. Among these, 49 patients were excluded as they did not fulfill the cephalometric condition (lacked cephalometric assessments, n = 20; elevated soft palate on imaging, n = 27; and other errors, n = 2). The final study population comprised 183 male patients stratified into two age groups which is middle-age and older patients. Figure 1 illustrates the flow diagram of patient screening and enrollment, including numbers at each stage.

An a priori sample size estimation to determine the minimum number of participants necessary to identify a statistically significant difference in AS between groups was conducted using G*Power 3.1 software, based on data from 6 middle-aged patients and 6 older patients (effect size d = 0.49, α = 0.05, 1 − β = 0.80). The required sample size was 107 middle-aged patients and 53 older patients.

This study was approved by the Ethics Committee of Osaka Dental University (ODU Med Ethics approval No. 111047) and was carried out in accordance with the Declaration of Helsinki.

### 2.3. Polysomnography

PSG was performed at external hospitals across multiple departments, including otolaryngology, respiratory medicine, and psychiatry, before patients were referred to our institution for mandibular advancement device (MAD) treatment. All PSG at external hospitals was performed according to the standard criteria [17,18]. Recordings comprised standard ones like electroencephalography, electrooculography, submental electromyography, electrocardiography, chest and abdominal respiratory impedance plethysmography, arterial oxygen saturation by pulse oximetry (SpO_2_), and nasal airflow with pressure transducer. Respiratory events were defined by the 2012 American Academy of Sleep Medicine criteria as follows: apnea was an attenuation of airflow by more than 90% for at least 10 s, and hypopnea was an attenuation of airflow by ≥30% or more for the same duration, if there was a desaturation of oxyhemoglobin by at least 3% or an arousal present. Additional parameters analyzed in this study included minimum oxygen saturation (MinSpO_2_), cumulative time with SpO_2_ < 90% (CT90), sleep efficiency, stage N1, N2, N3 sleep percentages, rapid eye movement (REM) sleep percentage, and arousal index.

### 2.4. Method and Measurement Conditions

We examined body mass index (BMI) as an index of obesity and cephalometric X-rays obtained for treatment purposes at our hospital’s Center for Dental Sleep Medicine.

### 2.5. Cephalometric X-Ray

**Figure 2:**
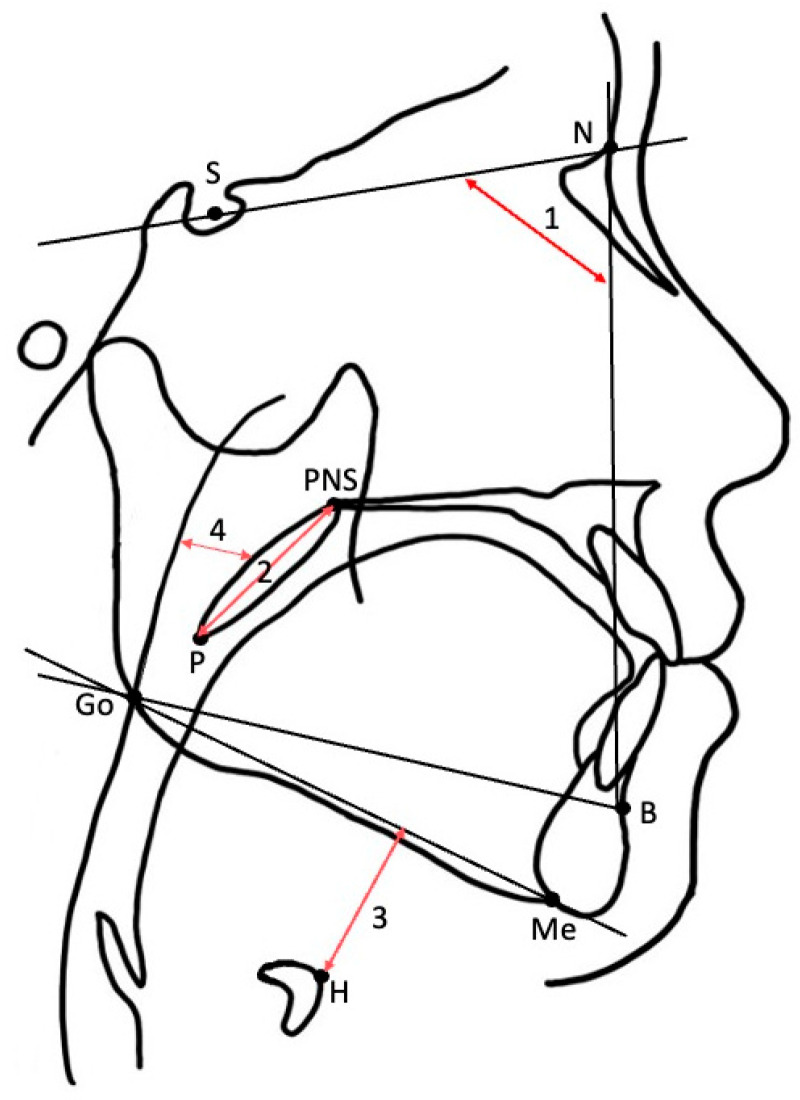
Cephalometric landmarks and measurements. (1) SNB; (2) PNS-P; (3) MP-H; (4) AS.

Cephalometric X-rays were obtained for clinical purposes as part of the standard diagnostic assessment of patients with OSA at the Center for Dental Sleep Medicine at our hospital. The lateral cephalometric imaging was performed with a CX-150WA high-voltage X-ray system (Asahi Roentgen Ind. Co., Kyoto, Japan) under standardized settings (80 kVp, 125 mA, 0.63 s). The patients were told to stand with their Frankfort horizontal plane parallel to the floor and with the central X-ray beam passing through the ear rods perpendicularly to the midsagittal plane [19]. All scans were recorded with their lips in normal closed positions, bite in intercuspal relation, and tongue and related musculature relaxed. We took an X-ray at the end of expiration [20]. Cephalometric analysis was performed as previously described (Table 1 and Table 2, Figure 2) [19]. To assess the reliability of cephalometric measurements, a random sample of 10 participants from the total sample was obtained. Each variable was measured independently by two authors, Liqin Wang and Kentaro Okuno, each performing one round of measurements. To evaluate the inter-rater reliability, we used the Intraclass Correlation Coefficient (ICC) computed with a two-way mixed effects model with the assumption of absolute agreement. The obtained ICCs were as follows: AS = 0.96; SNB = 0.94; PNS-P = 0.97; MP-H = 0.99. All of them were >0.90, which represents excellent inter-rater reliability in all the studied parameters. These procedures not only ensured measurement reliability but also served as part of our strategy to minimize potential bias in data collection.

### 2.6. Statistical Analysis

Levene’s test was used to examine the homogeneity of variances among groups. For normally distributed variables, independent sample t-tests were utilized to compare demographic characteristics (age, BMI), polysomnography indices (AHI, minimum SpO_2_, CT90, sleep efficiency, N1, N2, N3, REM sleep percentages, and arousal index), and upper airway morphology variables (AS, PNS-P, SNB, and MP-H) between middle-aged and older groups.

To investigate factors associated with OSA severity, multiple regression analyses were conducted in both the middle-aged and older groups, using AHI as the dependent variable. Based on the correlation matrix to account for multicollinearity, multiple logistic regression with the forced entry method was performed, including BMI, AS, SNB, PNS-P, and MP-H as independent variables. In addition, anthropometric and craniofacial variables were incorporated as covariates to adjust for potential confounders, particularly BMI. For all variables, effect sizes (β coefficients), 95% confidence intervals (CIs), and *p*-values were reported.

All statistical analyses were conducted using IBM SPSS Statistics (version 15.0), and we took a two-sided *p* < 0.05 to be statistically significant.

## 3. Result

A total of 183 patients were included in the analysis. Their mean age was 56.7 ± 0.83 years, and the mean BMI was 24.3 ± 0.24 kg/m^2^. The AHI was 29.0 ± 1.45 events per hour, and the MinSpO_2_ was 80.2 ± 0.62%. The CT90 averaged 3.7 ± 0.46%. The mean sleep efficiency was 74.4 ± 1.20%.

Sleep architecture analysis showed that patients spent on average 27.1 ± 1.21% of total sleep time in stage N1, 53.0 ± 3.39% in stage N2, 3.9 ± 0.41% in stage N3, and 15.6 ± 0.56% in REM sleep. The mean arousal index was 31.5 ± 1.40 events per hour. Cephalometric measurements demonstrated an average AS of 13.1 ± 0.27 mm, an SNB angle of 77.9 ± 0.47°, a PNS-P distance of 41.4 ± 0.43 mm, and an MP-H distance of 18.6 ± 0.53 mm (Table 3).

### 3.1. Polysomnographic and Cephalometric Characteristics by Age Group

Table 3 also summarizes all of the PSG, anthropometric, and cephalometric parameters of middle-aged and older patients.

There was no difference in AHI between middle-aged and older patients (29.5 ± 2.56 vs. 28.8 ± 1.76, *p* = 0.820). Older patients, nonetheless, yielded a significantly low percentage of REM sleep compared to middle-aged patients (13.5 ± 1.31% vs. 16.4 ± 0.59%, *p* = 0.047). Other PSG parameters, including MinSpO_2_, CT90, sleep efficiency, N1, N2, N3, and arousal index, were no significant difference between two groups.

According to anthropometric and cephalometric characteristics in the group of older patients compared with middle-aged patients, the BMI was lower (23.6 ± 0.45 kg/m^2^ vs. 24.6 ± 0.29 kg/m^2^, *p* = 0.049). AS was also higher in older patients (15.8 ± 0.52 mm) than in middle-aged patients (12.0 ± 0.27 mm, *p* = 0.000). No difference was found in SNB angle, PNS-P, and MP-H.

### 3.2. Multiple Regression Analyses

Multiple regression analyses were conducted to identify independent predictors of AHI within each age group in Table 4 and Table 5.

In the middle-aged group, BMI (β = 0.40, 95% CI: 1.46 to 3.41, *p* < 0.001), SNB (β = −0.18, 95% CI: −1.75 to −0.09, *p* = 0.030), and MP-H (β = 0.19, 95% CI: 0.10 to 1.01, *p* = 0.018) were significant independent predictors of AHI, while PNS-P and AS were not significant.

In the older group, no parameters were significant predictors of AHI.

## 4. Discussion

This study aimed to investigated age-related differences in craniofacial and upper airway morphology, and to compare anatomical factors contributing to apnea severity between older and middle-aged OSA patients, and found several novel findings related to OSA in older patients.

First, relative to middle-aged patients, older patients continuously had wider upper airway diameters. But even with morphologically larger airways, patients who were older still had OSA.

Secondly, anatomical variables significantly influenced AHI in middle-aged patients but did not significantly influence AHI among older patients, while most of the previous research highlighted anatomical narrowing as the main pathway to OSA [21]. Our results indicate that factors other than anatomical ones may be more determinant in disease pathogenesis in older adults.

### 4.1. Morphological Factors

The morphological characteristics of the upper airway, including PNS-P, AS, MP-H, and SNB, were compared between middle-aged and older patients. Among these, AS was significantly larger in older patients, whereas other structural indices were not significantly different between groups. These findings suggest that airway dimensions are well maintained or even widened among older patients.

Notably, our findings indicate a differential significance of anatomical factors between age groups. While in middle-aged participants, anatomical factors (BMI, narrow mandible, airway diameter) significantly influenced AHI, in older participants, anatomical factors did not significantly influence AHI. This might indicate that anatomical characteristics alone may not be used to determine the risk or severity of OSA in older individuals. Our findings support a previous study that reported that the increased prevalence of OSA among the geriatric adult population cannot be accounted for by changes in upper airway structure with aging [22].

All morphological indices for this study were measured with standardized lateral cephalometric radiographs, a commonly used and accepted method for assessing craniofacial and upper airway structure in OSA research.

### 4.2. Functional Mechanisms

Unlike during wakefulness, during sleep, narrowing of the airway is more likely to occur, and it has been reported that functional factors involved in maintaining the airway (loop gain, muscle responsiveness, and arousal threshold) play a role [23]. Based on the results of this study, it was considered that in awake OSA patients, the airway was larger in older adults than in middle-aged adults. Therefore, in older OSA patients, although a large airway is maintained during wakefulness, the function to maintain the airway is impaired compared to middle-aged adults, resulting in the airway narrowing at night and causing apnea.

Additionally, some studies suggest that fat distribution changes with aging, which may alter airway mechanics in a way that is not directly related to airway diameter [24,25,26]. Moreover, as people age, they are more likely to develop COPD, which is often accompanied by deteriorating sleep quality, such as increased sleep fragmentation and reduced slow-wave sleep [27]. These changes may impair respiratory regulation and exacerbate the severity of OSA. This hypothesis aligns with a previous study that reported that geriatric patients may have weaker pharyngeal dilator muscle function, which could lead to an increased collapsibility of the airway despite a larger anatomical space [3].

### 4.3. PSG Findings

Our PSG findings indicate that in comparison of age groups, only the REM sleep percentage was decreased in older patients, whereas sleep efficiency and proportion of N1 were not significant in the expected ways. These observations are supported by extensive evidence from aging physiology, which demonstrates that sleep becomes lighter, more fragmented, and less restorative with age [28,29]. Reduced circadian rhythm regulation is one of the fundamental mechanisms. Suprachiasmatic nucleus (SCN) degeneration in the hypothalamus with age has been found to weaken circadian timing, and this contributes to poor REM sleep and enhanced sleep fragmentation [30,31]. Dysregulation of circadian genes in the cortex and hypothalamus can further disrupt sleep–wake rhythm [32]. Neuroendocrine processes also make important contributions. Levels of melatonin decreased at night in later life, and this disrupts the onset and maintenance of deep and REM sleep [33]. Aging of the brain in areas like the hypothalamus and prefrontal cortex decreases the ability to maintain consolidated sleep, enhancing arousability and further disrupting sleep architecture [34].

### 4.4. Clinical Implications

The present findings have important clinical implications. Firstly, they indicate that screening for OSA among older patients cannot be based solely on anatomical risk factors such as BMI or craniofacial morphology. Our results suggest that even when airway morphology appears preserved or enlarged during wakefulness, impaired functional regulation (e.g., reduced ventilatory stability, neuromuscular responsiveness, or arousal threshold) may still lead to clinically significant OSA during sleep. Secondly, therapy needs to consider age-specific pathogenetic aspects. In middle-aged patients, treatment approaches targeting anatomical factors, such as weight reduction or surgical airway enlargement, have traditionally been applied as fundamental strategies. In older adults, however, in addition to addressing anatomical factors, therapeutic strategies aimed at improving functional mechanisms (ventilatory stability, neuromuscular activation, and arousal threshold stabilization) may provide additional benefit.

### 4.5. Limitations and Future Directions

This study has several important limitations that warrant consideration. First, potential selection bias must be considered, as all participants were recruited from a referral population seeking MAD treatment, which may disproportionately represent individuals with specific phenotypes or treatment-seeking behaviors. However, age distribution and disease severity within our cohort were similar to those within large epidemiological studies to support partial generalizability. This study focused on male OSA patients. In women, during the middle-aged period included in this study, factors such as menopause, which is a risk factor for OSA, and the effects of hormone therapy, which contribute to OSA reduction, are well known to be affected. For these reasons, in women, factors other than anatomical factors have a greater influence on OSA compared to men. Due to known gender differences in the pathophysiology, clinical symptoms, and treatment response of OSA, which particularly reduce external validity, this study excluded female subjects and was conducted exclusively with male subjects. Future research needs to include multi-center cohorts that are sex-balanced to enhance generalizability.

Second, although we adhered to STROBE reporting guidelines, there are inherent limitations to the retrospective cross-sectional design that include inability to determine temporal or causal relationships. The absence of longitudinal follow-up also precludes determining whether observed functional impairments are antecedents or consequences of aging and OSA severity. Prospective or longitudinal or multi-center studies will be necessary to establish temporal sequence of such relationships.

Third, although we applied strict exclusion criteria to minimize the influence of major comorbidities (e.g., severe cardiovascular, cerebrovascular, and neuromuscular diseases), residual confounding remains impossible to eliminate. Factors such as common cardiovascular diseases, diabetes, lifestyle habits (e.g., smoking, alcohol consumption), and medication use were not adequately controlled during analyses and could have affected OSA severity. In addition to this, we did not account for craniofacial developmental differences, ethnicity, or distribution patterns of body fat, all of which may markedly influence OSA severity and shape of the upper airway. These factors, along with other unmeasured confounders, should be considered in future studies to reflect more adequately the multifactorial nature of OSA.

Finally, measurement-related limitations should be acknowledged. Our reliance on polysomnography (PSG) parameters without direct mechanistic testing limits the strength of conclusions regarding neuromuscular factors. Although we considered cephalometric and polysomnographic parameters, we did not directly test for neuromuscular characters such as pharyngeal dilator responsiveness, arousal threshold, and loop gain. Particularly, we did not carry out direct physiological measurements such as electromyography (EMG) of airway dilator muscles, which could give more accurate assessment of neuromuscular dysfunction. In addition, oral cavity structures such as tongue volume, morphology of dental arches, and occlusion were not studied in our cephalic analysis, although they are known to affect upper airway collapsibility and OSA severity [26,35,36].

Together, these data suggest that functional rather than anatomical OSA severity contributors are more dominant in older patients. However, because lifestyle and co-morbidity may play equally salient roles as well, future studies need to control for such factors and include physiological as well as anatomical and behavioral variables to give a more complete characterization of age-related OSA phenotypes.

## 5. Conclusions

This study highlights distinct age-related differences in the pathophysiology of obstructive sleep apnea (OSA) between middle-aged and older patients. Multiple regression analyses demonstrated that in middle-aged patients, body mass index (BMI), mandibular position (SNB angle), and hyoid bone position (MP-H) were significant independent predictors of apnea–hypopnea index (AHI), whereas in older patients, no anatomical parameters were identified as significant predictors.

Although older patients exhibited larger upper airway diameters, they still experienced OSA-related sleep disturbances, and the underlying pathogenesis appeared to be more closely associated with functional decline rather than anatomical factors.

These findings have significant clinical implications. For middle-aged patients, therapy methods that aim to treat obesity and craniofacial deformities are still paramount. However, for older patients, therapy methods might place more emphasis on functional processes, for instance, reinforcing arousal threshold stabilization, advanced neuromuscular responsiveness enhancement, and sleep quality improvement, as against anatomical narrowing correction only.

## Figures and Tables

**Table 1:** Cephalometric landmarks.

Landmark	Interpretation
S	Midpoint of the fossa hypophysialis
N	Anterior point of the frontonasal suture
PNS	Most posterior point of the hard palate
B	Deepest anterior point in the concavity of the mandible
Go	Mid-plane point at the gonial located by bisecting the posterior borderline of the mandible
Me	Most inferior point of the mandible
H	Most antero-superior point of the hyoid bone
P	Most inferior tip of the soft palate

**Table 2:** Cephalometric measurements.

Measurement	Interpretation
Craniofacial skeletal	
SNB (deg)	Angle between the NSL and the line from B to N
Soft tissue	
PNS-P (mm)	Distance between PNS and P
Hyoid position	
MP-H (mm)	Linear distance between the mandibular plane and H
Airway	
AS * (mm)	Thickness of the airway behind the soft palate along a line parallel to the Go-B point plane

* Abbreviations: AS, airway space.

**Table 3:** Comparison of demographic, polysomnographic, and cephalometric characteristics between middle-aged and older participants.

Variables	All (N = 183)		Middle-Aged Group			Older Group				
	Mean	SE	N	Mean	SE	N	Mean	SE	t-Value	*p*-Value
Age (y)	56.7	0.83	129	50.4	0.51	54	71.7	0.69		
BMI (kg/m^2^)	24.3	0.24	129	24.6	0.29	54	23.6	0.45	1.98	0.049
AHI (events/h)	29.0	1.45	129	28.8	1.76	54	29.5	2.56	−0.23	0.820
MinSpO_2_ (%)	80.2	0.62	126	80.3	0.75	52	79.8	1.12	0.35	0.729
CT90 (%)	3.7	0.46	114	3.5	0.52	42	4.3	1.01	−0.76	0.448
Sleep Efficiency (%)	74.4	1.20	114	75.5	1.36	43	71.5	2.47	1.52	0.130
N1 (%)	27.1	1.21	115	26.3	1.40	43	29.1	2.38	−1.03	0.305
N2 (%)	53.0	3.39	115	54.3	4.55	43	49.4	2.68	0.64	0.524
N3 (%)	3.9	0.41	115	4.2	0.46	43	3.0	0.84	1.31	0.194
REM (%)	15.6	0.56	115	16.4	0.59	43	13.5	1.31	2.03	0.047
Arousal Index (events/h)	31.5	1.40	122	32.4	1.67	48	29.4	2.57	0.96	0.337
AS (mm)	13.1	0.27	129	12.0	0.27	54	15.8	0.52	−7.07	0.000
SNB (°)	77.9	0.47	129	78.4	0.34	54	76.7	1.37	1.71	0.088
PNS-P (mm)	41.4	0.43	129	41.1	0.37	54	42.2	1.14	−0.88	0.384
MP-H (mm)	18.6	0.53	129	18.2	0.61	54	19.6	1.04	−1.16	0.249

**Table 4:** Multiple regression analysis regarding variables that influence AHI in middle-aged group.

Variables	β	t-Value	95% Cl		*p*-Value
			Lower	Upper	
BMI (kg/m^2^)	0.40	4.93	1.46	3.41	0.000
AS (mm)	−0.08	−0.97	−1.53	0.52	0.334
SNB (°)	−0.18	−2.20	−1.75	−0.09	0.030
PNS-P (mm)	0.06	0.69	−0.49	1.02	0.492
MP-H (mm)	0.19	2.40	0.10	1.01	0.018

**Table 5:** Multiple regression analysis regarding variables that influence AHI in older group.

Variables	β	t-Value	95% Cl		*p*-Value
			Lower	Upper	
BMI (kg/m^2^)	0.27	1.95	−0.05	3.17	0.058
AS (mm)	−0.06	−0.42	−1.83	1.20	0.676
SNB (°)	−0.16	−1.06	−0.87	0.27	0.294
PNS-P (mm)	−0.09	−0.63	−0.83	0.44	0.532
MP-H (mm)	0.18	1.35	−0.22	1.11	0.184

## Data Availability

All data relevant to the study are included in the article.

## Abbreviations

The following abbreviations are used in this manuscript:

OSA	Obstructive Sleep Apnea
AHI	Apnea–Hypopnea Index
BMI	Body Mass Index
AS	Airway Space
PNS-P	Length of the Soft Palate
SNB	Sella-Nasion-B Point Angle (Mandibular Position)
MP-H	Hyoid Bone Position (Mento-Hyoid Distance)
